# Diversity, equity, and inclusion initiatives in the Medical Library Association: a look back at the last twenty-five years

**DOI:** 10.5195/jmla.2024.1967

**Published:** 2024-07-01

**Authors:** JJ Pionke, Thane Chambers, Marisol Hernandez, Brenda Linares, Beverly Murphy, Kelsa Bartley, Brandon T. Pieczko, Dean Giustini

**Affiliations:** 1 pionke@umich.edu, Instructor of Information, Syracuse University, Syracuse, NY; 2 thane@ualberta.ca Head of Research Impact Services, University of Alberta Library, Edmonton, Canada; 3 mhernan@cap.org Medical Librarian Specialist, College of American Pathologists, Northfield, IL; 4 blinares@umkc.edu Associate Dean of Library Services, University of Missouri at Kansas City, Kansas City, MO; 5 beverly.murphy@duke.edu Assistant Director at Duke University Medical Center Library Archives, Durham, NC; 6 k.bartley@med.miami.edu Librarian Associate Professor, Education and Outreach Librarian, University of Miami Miller School of Medicine, Miami, FL; 7 bpieczko@iu.edu Digital and Special Collections Librarian, Ruth Lilly Medical Library, Indiana University School of Medicine, Indianapolis, IN; 8 dean.giustini@ubc.ca UBC Biomedical Branch Library, Vancouver Hospital, Vancouver, Canada

**Keywords:** Diversity, equity, inclusion, history, retrospective, social justice

## Abstract

Over the past twenty-five years, the Medical Library Association (MLA) has pursued a range of diversity, equity, and inclusion (DEI) initiatives. This article, written by members of the *Journal of the Medical Library Association (JMLA)*'s Equity Advisory Group (EAG), outlines significant measures taken to raise awareness about specific concepts, opportunities, and challenges related to DEI among MLA members. Topics discussed include the impact of influential Black, Indigenous, and people of color (BIPOC) leaders, the establishment of DEI and social justice-focused membership communities, and specific initiatives led by various working groups and committees which have served to strengthen MLA's commitment to diversity, equity, and inclusion during the last three decades.

## INTRODUCTION

The Medical Library Association (MLA)'s Diversity and Inclusion Task Force conducted a member survey in 2019, which focused on the key demographics of our field and on attitudes towards diversity and inclusion among MLA members. Of those surveyed, a number of notable demographic patterns were revealed. For example, 72% of respondents were white and 79% were female [1]. The self-reported demographics within MLA resemble the membership of the American Library Association (ALA), whose own survey conducted in 2017 found that 86% of its respondents were white and 81% were female [2]. These statistics suggest that both librarianship generally and medical librarianship specifically need to increase their racial, ethnic, and gender diversity to more accurately reflect the demographics and represent the concerns of the communities they serve. Within MLA, there has been a concerted effort to be more equitable and inclusive in the range of its programs and committees, and to increase diversity among its membership. This article highlights some of the diversity, equity, and inclusion (DEI) initiatives that have taken place within the last 25 years within MLA. These include the election and impact of influential BIPOC leaders such as Beverly Murphy and Naomi Cordero Broering; the establishment of DEI and social justice-focused membership communities including the African American Medical Librarians Alliance, Accessibility and Disability Caucus, Latinx Caucus, and Social Justice and Health Disparities Caucus; and key initiatives led by working groups and committees including the Diversity Committee, *JMLA* Equity Advisory Group, and MLA Reads Virtual Book Discussion Group which have served to strengthen MLA's commitment to diversity, equity, and inclusion.

## INFLUENTIAL LEADERS

### Beverly Murphy, First African American MLA President

Beverly Murphy's trailblazing is exemplified by a series of firsts: first African American editor of the *MLA News* (2000), first African American chair of the Mid-Atlantic Chapter (MAC) of MLA (2002), first African American president since MLA's founding in 1898 (2018), and first African American recipient of the Marcia C. Noyes Award (2021). Despite the pressure and scrutiny placed on individuals when they are chosen to take on historically important roles, Beverly is widely-known for taking on these roles and challenges with ‘sass, elegance, pizzazz, and humor’ [3].

Beverly has played significant roles in MLA including working on the MLA Professional Recruitment & Retention Committee, MLA Diversity and Inclusion Task Force, *JMLA* Editorial Board and Equity Advisory Group, MLA Nominating Committee, and the MLA Board of Directors. Starting with each unique individual in mind and culminating in a shared vision, she also orchestrated the “I Am MLA” campaign, which grew out of the need for us to all gravitate towards the collective understanding that we are MLA and it is up to us, as members and volunteers, to do what needs to be done for our association. In addition to being a Noyes Award recipient, MLA's highest professional distinction, Beverly is an MLA Fellow, distinguished member of the Academy of Health Information Professionals, and was awarded by MAC with the Marguerite Abel Service Recognition Award and the Librarian of the Year Award.

Beverly has spent her entire career as a proponent of equity, diversity, inclusion, and belonging in librarianship. In her inaugural presidential address at MLA 2018, she said: “No matter what race we are, what color we are, what ethnicity we are, what gender we have, or whether we have physical issues—we are all information professionals, with a common goal, and that is to be an association of the most visible, valued, and trusted health information experts. Diversity drives excellence and makes us smarter, especially when we welcome it into our lives, our libraries, and our profession” [3]. Beverly's commitment to equity is not simply a bunch of words in a single speech. As coeditor of *Diversity and Inclusion in Libraries: A Call to Action and Strategies for Success* (Rowman & Littlefield, 2019), she helped to provide MLA with a framework and tools to build a profession where everyone has a role and can make significant contributions and positive changes. Beverly has helped to create an environment and culture where each of us is welcome to be ourselves while being responsible for our actions and words. This significant work was recognized when the North Carolina Library Association awarded her with the Roadbuilder's Award for Special Librarianship. Her work in librarianship will live on via the Beverly Murphy MLA Scholarship for Underrepresented Students which awards up to $5,000 to a student who shows excellence in scholarship and potential for accomplishment in health sciences librarianship.

### Naomi Cordero Broering, First Latinx MLA President

Naomi Cordero Broering (1929–2023) was the first Latinx person to serve as MLA president (77th president, 1996–1997). She was the 21st editor of MLA's *Bulletin*, and in 2003, received MLA's highest honor, the Marcia C. Noyes Award. Born in New York City to Puerto Rican parents, Broering and her family later relocated to California. She was raised both bilingual and bicultural, and excelled in her outreach to diverse populations in settings serving BIPOC, and most importantly, communities with a high incidence of HIV.

As MLA President, Broering identified five priorities with both short-term and long-term objectives: education and distance learning, membership development, research project for the creation of MLANET, electronic publishing, and advocacy for the profession [4]. Broering sought to improve MLA's capabilities in education and distance learning to expand continuing education opportunities for lifelong learning to MLA members, while leveraging existing technologies. Expanding MLA membership was a significant endeavor as well, paving the way for a newer generation of information professionals. Under Broering's leadership, MLA added over three hundred new members to the association. During Broering's MLA Presidency, there was an expansion of MLANET's capabilities, development of electronic publishing of MLA publications, and advocacy for the library profession at meetings and events held by organizations like the American Hospital Association and the National Alliance for Caregiving, among others [4].

Broering leaves an enduring legacy in multiple roles as librarian, scholar, collaborator, visionary, advocate, prolific author, and avid supporter of MLA and the National Library of Medicine (NLM), as well as being the first Latinx person to lead MLA.

## COMMITTEES, COMMUNITIES, AND WORKING/MEMBER GROUPS

### From DEI Task Force to Diversity Committee

DEI initiatives were brought to the forefront of MLA when the Board of Directors decided to prioritize DEI as a strategic goal. Announced in December 2016 by Teresa L. Knott, AHIP (President, 2016–2017) and solidified as a goal by the Board in May 2017, Barbara A. Epstein, AHIP, FMLA (President, 2017–2018) issued a call for the formation of a Diversity and Inclusion Task Force (DITF) to actualize MLA's Diversity and Inclusion strategic goal. Chaired by Sandra G. Franklin, AHIP, FMLA, the DITF operated from September 2017 to May 2020 and included 12 additional members, along with MLA staff liaison, Tomi Gunn [5]. The DITF's aim was to “evaluate and improve MLA practices as they relate to diversity and inclusion,” which led to the development of five goals to guide the task force's work:

1. Build activities and programs that create and sustain diverse, inclusive, and welcoming cultures and practices;
2. Ensure that members, volunteers, and staff have a high level of awareness of issues related to diversity and inclusion;
3. Ensure that what we do as an organization, and how we do it, reflects the essential values of diversity and inclusion;
4. Attract a diverse community of members that reflects the diversity of the profession and those we serve; and
5. Apply the best practices of professional associations with regard to diversity and inclusion [5].

The DITF collaborated with MLA members and leadership on various activities, conversations, initiatives, and made key recommendations, many of which were implemented during the time of the DITF and continue to influence the structure and work of MLA. Significant changes resulting from recommendations made by the DIFT include:

- Review and revision of MLA's vision, mission, values, and code of ethics and changes to language throughout all MLA documents, for example, changing Hispanic to Latinx. The motions were accepted and passed by the MLA Board on September 3, 2019 [5, supplemental Appendix D].
- Contribution to the Communities Transition, which involved collaboration with the Communities Task Force and MLA leadership to create a more inclusive professional experience for membership. Two significant recommendations approved by the MLA Board in 2018—replacing the dual-level of MLA member communities (sections and special interest groups) with a single tier (caucuses) and eliminating the financial barrier to joining an MLA member community by doing away with community dues–were implemented with other changes in September 2019, with an immediate positive effect on MLA's diversity and inclusion [5].
- The Diversity and Inclusion Task Force 2019 Survey was the first of its kind for the organization. In October 2019, the online survey administered to the membership revealed the demographics of the association and gave more clarity about how members feel about MLA as well as the DEI efforts of the association [1].

Both the MLA Diversity and Inclusion Task Force Report [5] and the Diversity and Inclusion Task Force 2019 Survey Report [1] document many more initiatives and provide deeper insights into the transformative work of the Task Force.

In 2020, the DITF became the MLA Diversity, Equity, and Inclusion (DEI) Committee, led by Xan Y. Goodman, AHIP, the committee's first chair. The MLA Diversity, Equity and Inclusion (DEI) Committee is now the coordinating and advisory body that evaluates and seeks to improve MLA practices and programs as they relate to DEI. The committee aims to:

- promote and encourage a diverse MLA leadership at all levels;
- be a voice for DEI within MLA;
- advise and collaborate with MLA communities and committees on DEI-related issues;
- encourage, recommend and contribute to DEI-related programs, events, and resources for MLA members and the public;
- recommend strategies to increase diversity in the profession; and
- lead special DEI-related projects as directed by the Board of Directors [6].

The DEI committee continues the work of the original task force in many ways, such as:

- The Living Library Program was established by original DITF member Amy Taylor for members to learn about each other's diverse life experiences that can lead to understanding and greater connections through sharing these stories in a safe space.
- Collecting and disseminating diverse conference programming and learning opportunities offered by MLA.
- Implementing an infrastructure to gather what various MLA Committees are doing to promote DEI and facilitate collaboration, to gather information related to DEI best practices and information, and to inform MLA members about DEI committee activities.

### African American Medical Librarians Alliance (AAMLA)

The African American Medical Librarians Alliance (AAMLA) is an affinity group within MLA formed in response to the necessity for a space where information professionals from historically marginalized communities could unite and ensure their representation within the organization. Before 2000, African American information professionals within MLA relied on informal communication throughout the year to foster collaboration and networking, typically culminating in social gatherings like dinners and other activities during MLA conferences. After years of social networking, it became apparent that strategic development within the Association was essential for advancement and success in a field predominantly occupied by white women. After meeting the criteria to form as a Special Interest Group (SIG), the African American Medical Librarians Alliance SIG was officially recognized in 2000 at the Annual MLA meeting in Vancouver, Canada.

Today, the AAMLA Caucus of MLA stands committed to bringing the challenges and issues of historically excluded information professionals to the forefront. The caucus’ priorities include cultivating opportunities for recruiting and retaining diverse librarians and information professionals, mentoring and leadership, developing expert skill sets, and increasing engagement within MLA. AAMLA's role is to help all medical information professionals understand and appreciate the dynamics of cultural diversity, as well as recognize and address the needs for cultural competence and humility in healthcare environments. In accomplishing these goals, AAMLA ensures that the efforts, achievements, and accomplishments of African American MLA members are highlighted as a personal identity of AAMLA. Membership in AAMLA is open to all members of the Medical Library Association and is currently composed of information professionals who are largely employed at academic institutions, hospitals, and community colleges throughout the United States.

#### Accessibility and Disability Caucus

With the formation of the DITF in 2017 under Sandra G. Franklin, there was an awareness of the concerns of marginalized groups, including people with disabilities. On the Task Force, JJ Pionke was the voice of disability and accessibility. When the Annual Meeting Innovation Task Force was formed in 2018, JJ was the liaison between the two task forces and an advocate for improved accessibility of the annual meeting. MLA efforts toward meeting accessibility improvements have included a quiet room for meditation and sensory safe place when experiencing sensory overload, alternative quiet activities like coloring and board game night, and a portable walking maze for meditation. JJ spearheaded the Accessibility and Disability Caucus in 2019, which has continued JJ's work through various activities including educational and outreach to both the membership and at the annual meetings.

### Latinx Caucus

In 2014, the MLA Latinx Caucus was established and was known initially as the Latino Special Interest Group. In its first year, the group's two co-conveners, Brenda Linares (MLA president-elect, 2024–2025) and Diana Almader-Douglas, led the collaborative effort with other Latinx health sciences librarians and MLA members. It was the second affinity group of MLA, after AAMLA, and established to lead efforts to acknowledge and address issues related to, and affecting, the Latinx community. In 2024, the Latinx Caucus is celebrating its 10-year anniversary. Over those first ten years, members have been involved in diverse projects that benefit health sciences librarians and the Latinx community. In 2023, the Latinx Caucus deservedly received the Caucus Project of the Year Award for their *Hispanic/Latinx Inclusive Terminologies Project*, where the caucus addressed a need to review terminology and capture the diversity of the Hispanic/Latinx populations in the United States. The award serves as a vehicle to highlight collaboration and innovation amongst health sciences librarians [8].

Latinx Caucus members have been successful in collaborating on projects with other MLA members and caucuses, as well. These initiatives include Covid-19 Spanish Language Resources, a guide for health professionals, patients, and the public; Hispanic/Latinx MESH Terms, recommendations for changes to the current terminology related to the Hispanic/Latinx populations; and recommendations for devising Hispanic/Latinx Search Hedges for optimal search results while searching the biomedical and health literature [7]. Currently, the Latinx Caucus works via various task forces to address programming, member engagement, outreach, research, and scholarship. Its long-term goals involve expanding BIPOC representation in the field of health/medical librarianship, increasing opportunities for caucus engagement, and forming bridges with library colleagues across Latin America.

### Social Justice and Health Disparities Caucus

The Social Justice and Health Disparities (SJHP) Caucus has made many contributions to MLA's DEI and social justice initiatives over the past 25 years, building on efforts that began more than 50 years ago. Its origins can be traced back to 1972, when Jerome S. Rauch of the University of Pennsylvania and other MLA members submitted a petition to form a “Relevancy Group,” later known as the Relevant Issues Section of MLA [9]. Its purpose was, “to promote the educational, scientific, and professional growth of its members with emphasis on social issues” [10].

In its early years, SJHP's “Relevant Issues Section Bibliographies” columns published in *MLA News* served as an important vehicle to inform MLA members about developing social issues and trends that could impact their professional lives, including occupational health (1985), hospice care (1987), treatment of HIV/AIDS patients (1989), workforce diversity (1991), alternative medicine (1995), and advance directives (1997). The group has collaborated with allied groups such as the LGBTQIA+ and Health Disparities Caucuses to offer programs at MLA annual meetings and sponsored timely MLA resolutions on social issues including the medical consequences of nuclear war (1983), anti-Apartheid efforts in South Africa (1986), AIDS (1987), addressing the health care needs of vulnerable populations (1997), and global violence (1996) [9].

In 2018, the Relevant Issues Section was renamed the “Social Justice Caucus” and its primary concern became “social justice issues that have an impact on how health sciences librarians perform their roles” [9]. Two years later, the Social Justice Caucus merged with the Health Disparities Caucus to become the “Social Justice and Health Disparities Caucus” [9]. The purpose was to promote, “the educational, scientific, and professional growth of its members with emphasis on social issues that have an impact on how biomedical librarians perform their roles,” and to, “serve as a resource for MLA members related to health disparities and health inequities, promote awareness of literature and data related to social justice and health disparities, and identify collaboration for education and programming to other MLA caucuses” [11].

In June 2020, the Social Justice and Health Disparities caucus continued to demonstrate its nearly half-century commitment to social justice and DEI initiatives by issuing a statement of solidarity with the efforts of Black Lives Matter movement protesters in response to the murder of George Floyd and to the continued police violence towards Black Americans, which the caucus identified as, “one of the many detrimental health disparities our caucus organizes to address” [12].

### *Journal of the Medical Library Association's (JMLA)* Equity Advisory Group

Partially as a result of events surrounding George Floyd's murder in 2020, *JMLA* created an Equity Advisory Group (EAG) to examine ways of better incorporating DEI into its policies, procedures, and practices. The EAG strives to provide more opportunities for members of the Black, Indigenous, and people of color (BIPOC) community; LGBTQIA+ community; and for people with accessibility needs to serve as authors, reviewers, and editorial team members. Since the EAG was formed, *JMLA* has examined a range of existing, structural challenges in adopting DEI-informed practices, including how to respond to a racist incident involving one of *JMLA's* copy editors in 2020, and academic integrity concerns regarding a section editor in 2023.

Since 2020, the EAG has engaged in projects such as increasing diversity among the editorial board; revising the editorial style guide to be more inclusive; creating a name change policy for previously-published *JMLA* authors who want to change how their name appears; developing a DEI training program for the editorial board and reviewers; and other initiatives including gathering DEI-related demographic data for editorial board members, reviewers, and authors.

The EAG will conduct a DEI survey of reviewers in 2024 in order to improve recruitment of authors from historically marginalized communities. Other future projects will include developing a first-time author mentoring program and improving article submission, editorial workflows, and other accessibility-related aspects of the publication process.

### MLA Reads Virtual Book Discussion Group

The MLA Reads Virtual Book Discussion Program grew from “Transforming Libraries Using Implicit Bias Training,” a special content session held at the MLA 2018 Annual Meeting. In that original session, participants conveyed the need for safe spaces to gain knowledge, converse with others, and to confront the implications of biases on their work and personal lives. Shannon Jones and Kelsa Bartley, original organizers of the 2018 special content session, planned and facilitated the first virtual book discussion for approximately fifty librarians on the topic of implicit bias, using Mahzarin R. Banaji and Anthony G. Greenwald's book, *Blindspot: Hidden Biases of Good People*, as a platform for safe and thought-provoking interactions for discussion on challenging issues and topics in a safe, welcoming, and inclusive environment [5].

The MLA Reads program is now in its 6th year; member facilitators have led over 700 members of MLA and other non-member librarians across the country through important conversations in safe, virtual spaces, about a wide range of topics related to diversity, equity, inclusion, and ability. The program has inspired offshoots, such as the AAMLA Reads discussions facilitated by the African American Medical Librarians Alliance Caucus (AAMLA) and the AAHSL Reads Virtual Book Club facilitated by the Association of Academic Health Science Libraries (AAHSL) DEI Committee. Facilitators Shannon Jones, Kelsa Bartley, Melissa DeSantis, Ryan Harris, Don Jason, and Dede Rios also wrote a book chapter highlighting the importance of having conversations about DEI and ability in libraries; providing details on how and why Banaji and Greenwald's book was used to discuss the topic of implicit biases and the harms they can cause; and how the book discussion program became a catalyst to advance discussion of difficult topics. The chapter provides details about discussion group organization and logistics, facilitator training, how the program is evaluated for improvements, and lessons learned during the course of the program's existence [13].

## LOOKING TO THE FUTURE

While much progress has been made in the area of DEI within MLA, there is still much to do. The library profession as a whole is predominantly white and female, and these tendencies are no different in medical librarianship. Diversification of the profession, which includes recruitment and retention of people from historically marginalized communities, needs to be a major priority for both the profession and for MLA. Representation matters. We need to continue our self-examination of our policies, attitudes, and goals to be more diverse, inclusive, and equitable. While we need to look back and understand our history and how it has negatively affected our colleagues from diverse backgrounds, we also need to look forward to how we build a better MLA that truly values and includes all voices.
